# Supplementary material for: Urinary Volatile Organic Compound Testing in Fast-Track Patients with Suspected Colorectal Cancer
Source: Cancers (Basel). 2022 Apr 24;14(9):2127. doi: 10.3390/cancers14092127 (PMC9099958; doi:10.3390/cancers14092127)
Supplement: Supplementary file 1 [file cancers-14-02127-s001.zip › cancers-1653845-supplementary.pdf]

Supplementary Materials

Urinary Volatile Organic Compound Testing in Fast-Track Patients with Suspected Colorectal Cancer

1. Supplementary Material Appendix A—Overview of the Instrumentation Used in The DISCOVER Study

During the DISCOVER study, urinary volatile organic compounds were analysed using three methods;

1. Automated Thermal Desorption – Gas Chromatography Mass Spectrometry (GC-MS)
2. Selected Ion Flow Tube – Mass Spectrometry (SIFT-MS)
3. Field Asymmetric Ion Mobility Spectrometry (FAIMS)

1.1. Gas Chromatography—Mass Spectrometry

GC-MS is considered the gold standard method for volatile organic compound analysis. In the chromatography phase VOC molecules are physically separated based on chemical properties such as boiling point and electrical charge. These molecules are then ionised with an electron beam and fragmented allowing qualitative and relative quantitative analysis of complex matrices. Each compound provides a spectra of ionized fragments (also known as daughter ions), this spectra is unique for each compound and by comparison to a library of known compound spectra (the national institute of standards and technology (NIST) library) a compound ID is made.

The instrumentation used here was as follows:

i. An adapted SRI instrument single sample headspace autosampler for sample incubation and loading of the thermal desorption tubes.

ii. Two bed thermal desorption tubes (Tenax and Sulficarb, Markes International, Bridgend, UK).

iii. Thermomatrix150 Automated Thermal Desorption instrument (Perkin Elmer, Bucks, UK)

iv. Perkin Elmer Clarus 500 Gas Chromatography-Mass Spectrometry (Perkin Elmer, Busks, UK).

v. Gas Chromatography column – Phenomenex ZB-624 (60 metre, 0.25mm internal diameter, 1.4 micron film thickness) (Phenomenex, Cheshire, UK).

Sampling Method

The frozen urine was placed into a water bath for 30 minutes at 60°C. A nitrogen purge gas (99.998% purity BOC UN1066) was pushed into the sample containing headspace vial for two minutes at 80 mL/min, the outflow from the headspace vial passes through the thermal desorption tube. The desorption tube was then transferred immediately to the Automated Thermal Desorption instrument. The thermal desorption method has been published previously (Gould, O., de Lacy Costello, B., Smart, A., Jones, P., Macmaster, A., Ransley, K., Ratcliffe, N. Gas chromatography mass spectrometry (GC-MS) quantification of metabolites in stool using 13C labelled compounds. Metabolites. 2018 Dec; 8(4):75.).

The obtained data files were first converted into .cdf files for analysis using the online XC-MS statistical program. This program carries out analysis to find significant differences between the clinically relevant groups. For comparison of two groups unpaired non-parametric (Mann-Whitney) tests were used, for comparison of all three groups Kruskal-Wallis non-parametric tests were used. This primary analysis formed the basis for subsequent statistical analysis.

1.2. Selected Ion Flow Tube—Mass Spectrometry (SIFT-MS)

SIFT-MS is a quantitative mass spectrometry technique providing real-time measurement of VOC concentrations. It is based on chemical ionization induced by precursor ions $H3O^+$, NO^+ and $O2^+$ generated in a microwave discharge and selected by a quadrupole mass filter. However, for the quantification of compounds to be achieved specific target analytes must be known. To analyse the complex unknown matrix of urinary VOCs the SIFT-MS was run in full scan mode through a defined mass range (0–200 units in this case) providing an output of ion count over mass-to-charge ratio (*m*/*z*). This data can be used to compare clinically relevant groups and identify differences.

SIFT-MS instrumentation was as follows:

i. Voice 200 SIFT-MS (Anatune, Cambridge, UK)).

ii. SIFT-MS heated inlet extension sampling head designed for direct headspace/breath measurements (Anatune, Cambridge, UK).

iii. Nalophan bags (Parker's food Machinery Plus ltd, Bognor Regis, UK)

Sampling Procedure

The urine sample was first thawed in a water bath at 40 °C for 5 min until just thawed. Then 2.5 mL of urine was transferred to a nalophan bag and filled with hydrocarbon free air (100% synthetic air 287478-L-C from BOC Ltd, UK) the bag was then placed in a drying cabinet also at 40 °C for a further 25 min. The passive flow of the SIFT-MS was used to draw in the samples from the bag at a rate of 28 mL/min.

Each day prior to use, the SIFT-MS calibration method was performed, and all pressures and flows recorded to ensure they fell within tolerance. The calibration method checks all the internal pressures and flow and quadrupole alignment and detector linearity. The SIFT-MS was run in full scan mode which detects the mass to charge ratio (*m*/*z*) for VOCs across a selected range 0– 200 in this case. The measurement limit for each mass unit was set to 100ms or 10000 counts, whichever is reached first. The total scan time per sample was 300 s, with 0 s flow measuring. The SIFT-MS in full scan mode scans H_3O^+ and O_2^+ reagent ions sequentially over the course of the sample run; this parameter cannot be altered. A blank sample bag filled only with the hydrocarbon free air was analysed between each sample using the same method for reference.

In this mode the SIFT-MS has limited quantitative and quantitative ability and instead provides an output of m/z versus ion count which gives a distinct profile for each sample. As the SIFT-MS uses a soft chemical ionisation, knowing the *m*/*z* of the ions present can provide clues towards the possible compounds in the sample.

1.3. Field Asymmetric Ion Mobility Mass Spectrometry

FAIMS is a type of ion mobility spectrometer designed to measure mobility in different strength electric fields. The instrument operates using air as the carrier gas and at room pressure and temperature. However, it does not identify specific chemicals and a "machine learning" like approach is normally applied. In use, the sample/VOC enter the instrument and is ionized using a radioactive Ni63 source. The resultant ions are passed through a comb-like structure to which an alternative electric field is applied, consisting

of a short high potential and a longer low potential, but with time x potential being equal. In this field, the ions are attracted, repelled, or not affected. A compensation voltage is applied to remove the drift, thus by scanning through a range of compensation voltages and field strengths a mobility map of the sample is obtained. FAIMS has very high sensitivity and has been used extensively for security applications, but more recently have repurposed for medical applications including CRC detection [vi]. In this study a commercial FAIMS unit was used (Owlstone Lonestar™). This unit was fitted with an ATLAS sampling system (Owlstone Medical, Cambridge, UK).

Sampling Procedure

Urine samples were prepared at the point of collection. In this case 5ml of urine sample was aliquoted into a 10 mL glass vial and sealed at that time. For analysis, the vials were defrosted in a water bath at 40 °C for 30 min. Once defrosted, the lid of the vial is removed and the whole vial is placed inside the ATLAS sampling system. The ATLAS was powered for 30 min before the beginning of the experiments and the chamber temperature set to 40 ± 0.1 °C. The sample was heated for a period of 10 min, with no air flow, to generate a headspace. A flow rate of 200 mL/min of synthetic air (287478-L-C, BOC Ltd, UK) was put over the sample, with a further 1.8 L of synthetic air added as make-up air. This was then introduced into the machine as a continuous flow. Each sample was tested three times sequentially, with each test taking around 60 s. The FAIMS was scanned from 0% to 99% dispersion field in 51 steps, −6 V to +6 V compensation voltage in 512 steps and both positive and negative ions were detected to create a test file composed of 52,224 data points. A blank sample (empty vial) was used at the beginning of a batch of samples and in between each sample to reduce carry-over between samples and to ensure that the instrument was clean. This was also used as a quality control check to ensure that the RIP (reactive ion peak) was of an appropriate intensity and path.

2. Supplementary Material Appendix B—Data Analysis

2.1. GCMS and SIFT-MS Analysis

The GC-MS and SIFT-MS output data was square root transformed. This square root variance stabilising transformation minimises the impact of outliers which might otherwise adversely exert undue influence on model validity and minimise the over capitalisation on chance sample idiosyncratic features which might not generalise to new data. Individual sample features were screened for statistical significance using the non-parametric Mann Whitney Wilcoxon rank sum test and the parametric Welch (separate variances) *t*-test to ensure modelling was only performed using potentially useful markers. Potential markers with a high degree of information overlap may be redundant and their joint inclusion in a model may result in models with a high degree of variance. We therefore sought to use a small subset of potentially useful square root transformed features, avoiding problems associated with multicollinearity, to model the three disease groups without overly populating the number of features to disease group ratio to help avoid model overfit. Imposing a condition of linearity between markers and predicted disease group may be overly restrictive and for this reason two-layer artificial neural networks were used to develop proof-of-concept classifiers for the GC-MS data and the SIFT-MS data in all cases partitioning the data 70% to 30% for training and testing during model development. All models were run 10 times to avoid selection bias. Sensitivity, specificity and Area Under the ROC curve (AUROC) were used to summarise models.

2.2. FAIMS Analysis

The output from the FAIMS analysis produces a high dimensional dataset, which comprises over 50,000 data points per sample, thus the pipeline applied is different to that used for GCMS and SIFT-MS. First, a threshold was applied to the data, to zero data points that hold no chemical information. This threshold is calculated from the background value, with a small addition added. The same threshold was applied to all the samples and was twice the average value of the background. Then 10 fold cross validation was

used, with the data split into 10 groups, with 9 groups being used for training with the 10th group being the test set. This was repeated 10 times until all the data had been a test sample. Within each fold, a rank sum test was applied to each datapoint across all the training samples and the 50 data points with the lowest p-value were then used to train the models (specifically Neural Network and Random Forest) and these models were then applied to the test set. Statistical parameters were then calculated from the resultant probabilities, as with the GCMS and SIFT-MS data.

3. Supplementary Material Appendix C—VOC Annotation

Table S1. Compounds identified by XC-MS as being altered in the colon cancer group vs controls and/or polyps. The specific *m*/*z* values were identified by XCMS along with the corresponding retention times. A library search was undertaken using the NIST mass spectral database and only compounds with a forward and reverse match >800 with a high probability were named. The mass spectra and library spectra are provided below for each compound.

Retention Time/Min	Compound Annotation	Change in Cancer Group vs. Polyps/Control	Studies Previously Reporting this VOC in Urine Linked to Cancer
1.67	Carbon disulphide	Increased	Reported in CRC/Breast cancer [1]<br>Increased in lung cancer [2]
2.07	Acetone	Increased	Increased in CRC [3]<br>Increased in GI cancer [4]
3.00	Ethanol	Increased	Increased in GI cancer [5]
3.19	Unknown (NIST library best match for 2,2,6,6-tetramethyl-4-ethyl-heptane)	Increased	-
4.92	Dimethyldisulphide	Increased	Increased in head and neck cancer [6]<br>Reported to decrease in CRC [7]
5.79	m-xylene	Increased	p-xylene increased in prostate cancer [5]
5.86	4-heptanone	Increased	Increased in breast [8] and bladder cancer[7]<br>Decreased in lymphoma and renal cell carcinoma [7]
8.83	Benzenethiol	Increased	Not identified previously in studies of VOCs linked to cancer
12.35	Pyrrole	Increased	pyrroles were increased in a range of cancers including CRC [9]
17.77	1,6-dichloro-1,5-cyclooctadiene	Increased	Not identified previously in studies of VOCs linked to cancer
18.92	Biphenyl	Increased	Not identified previously in studies of VOCs linked to cancer
19.06	Phenol	Increased	Increased in CRC/breast cancer [7]
21.95	dibenzofuran	Increased	Benzofuran derivatives linked to cancer but not identified previously in studies of VOCs linked to cancer

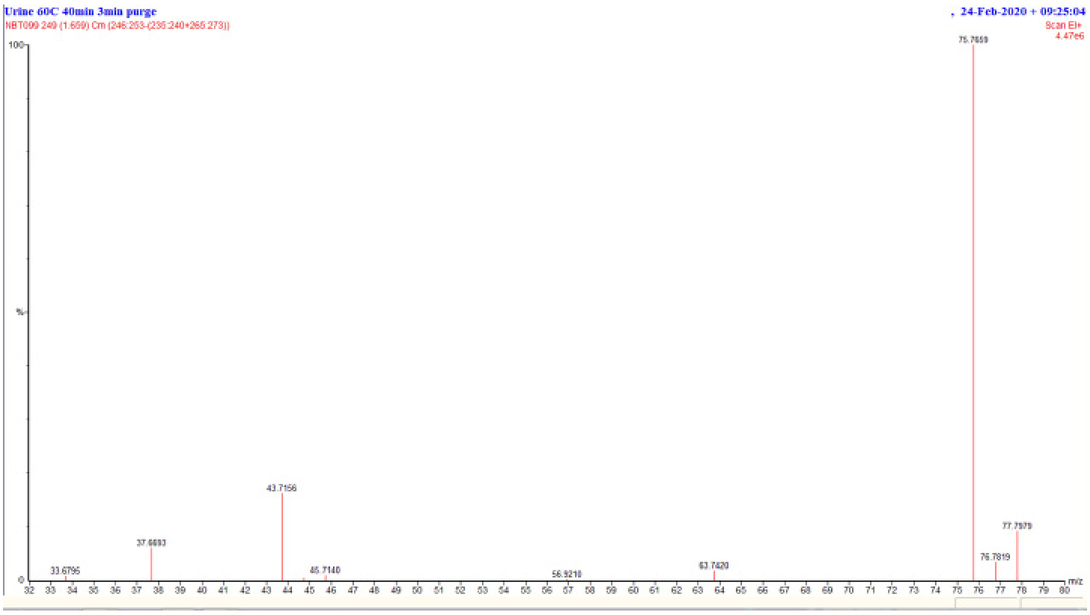

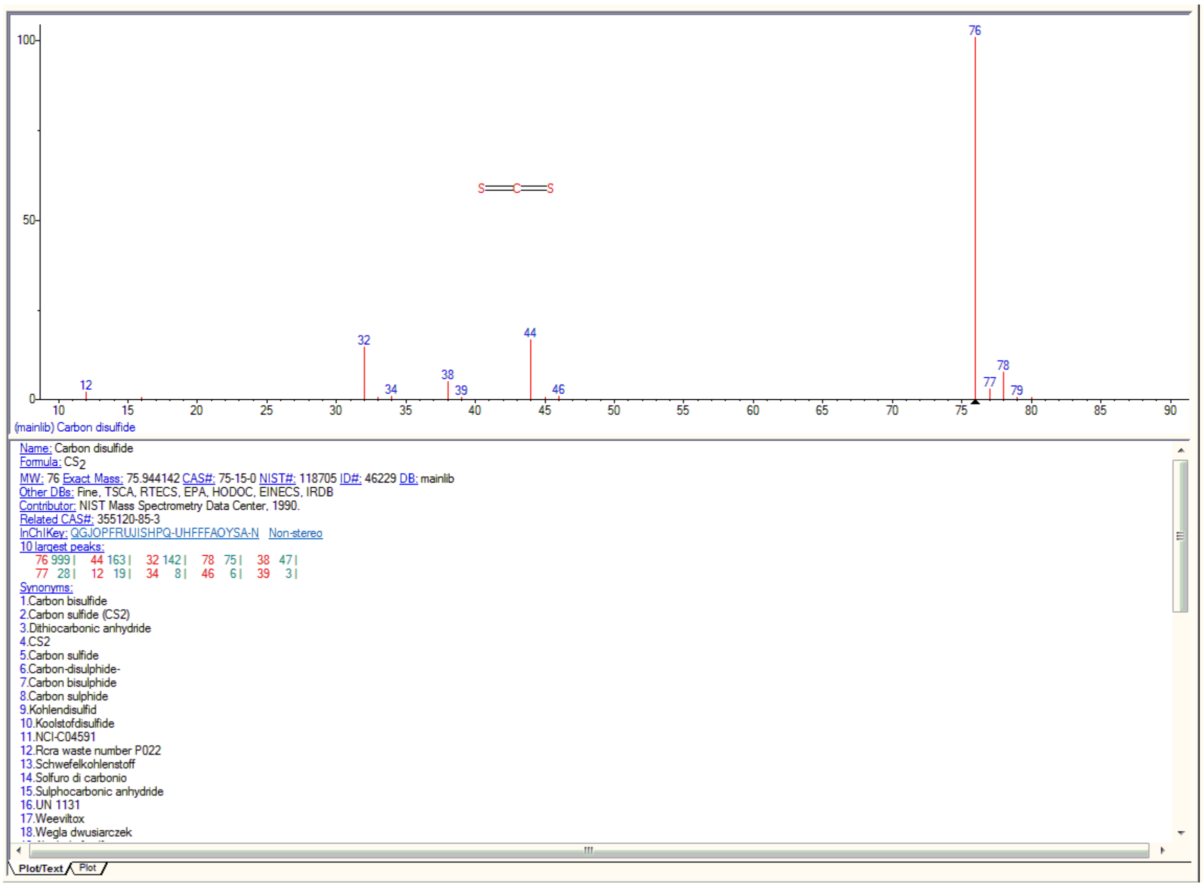

Figure S1. Experimental mass spectra and library spectra for compound annotated as Carbon disulphide.

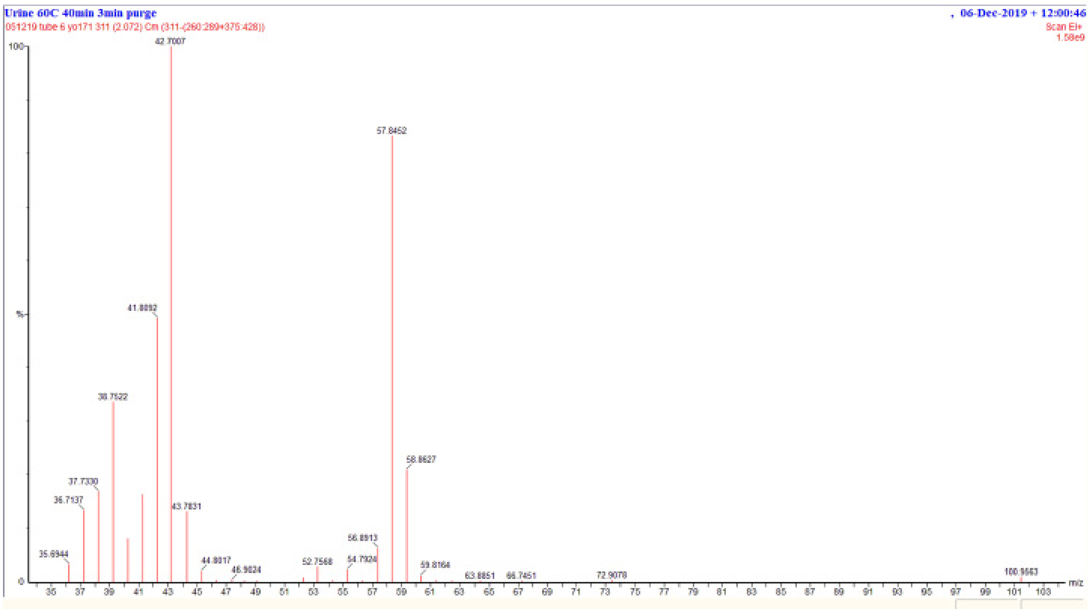

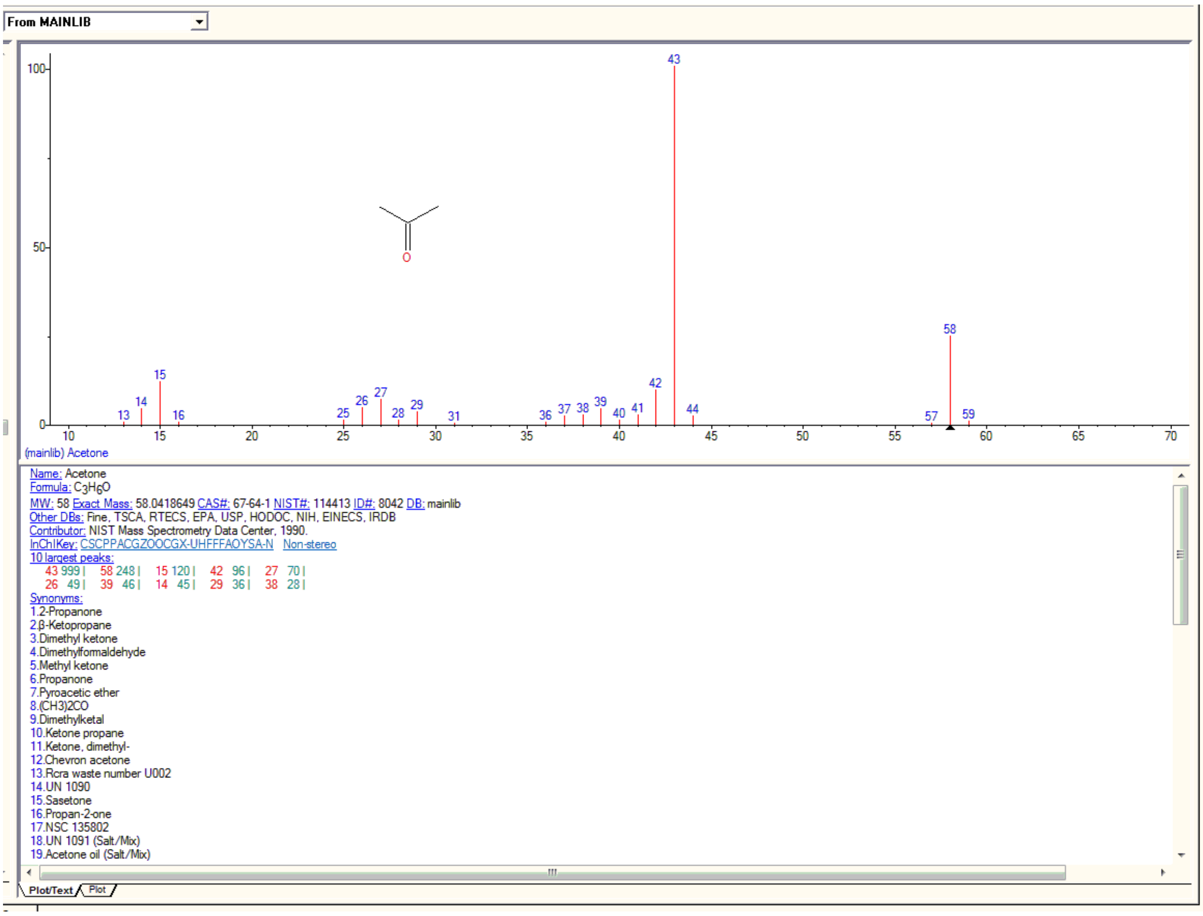

Figure S2. Experimental mass spectrum and library spectrum for compound annotated as acetone.

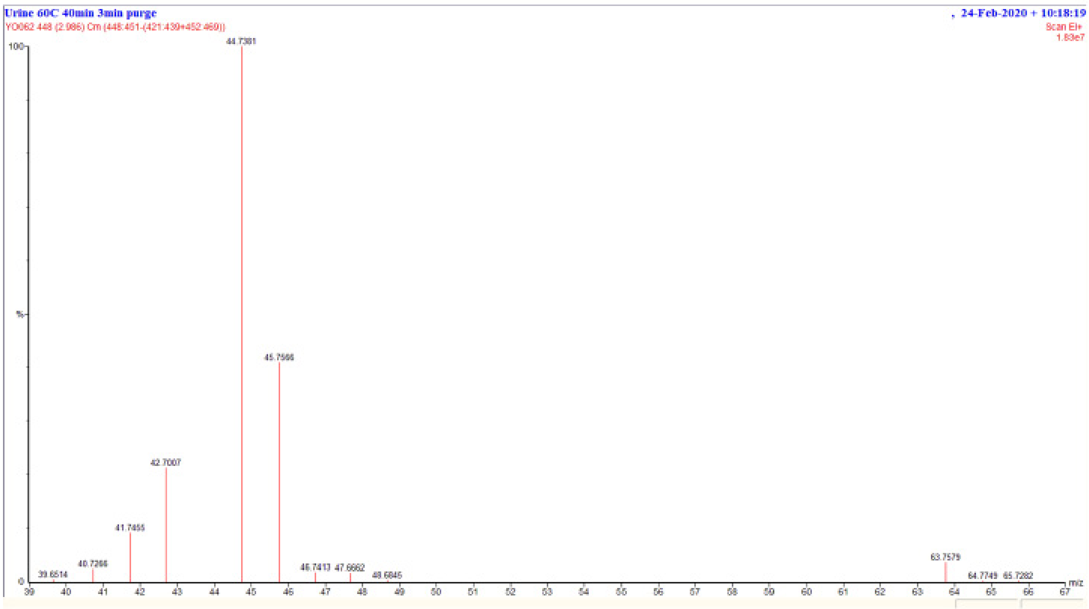

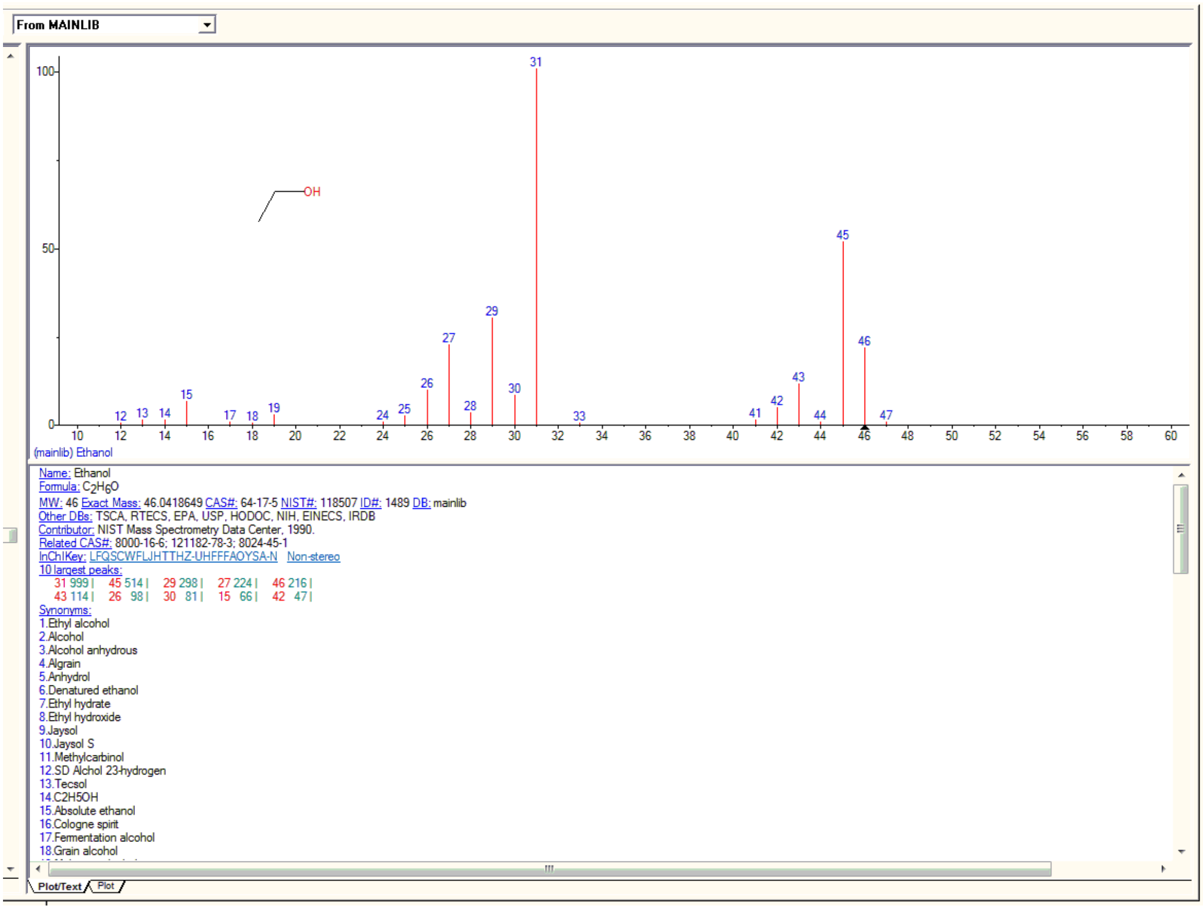

Figure S3. Experimental mass spectrum and library spectrum for the VOC annotated as ethanol.

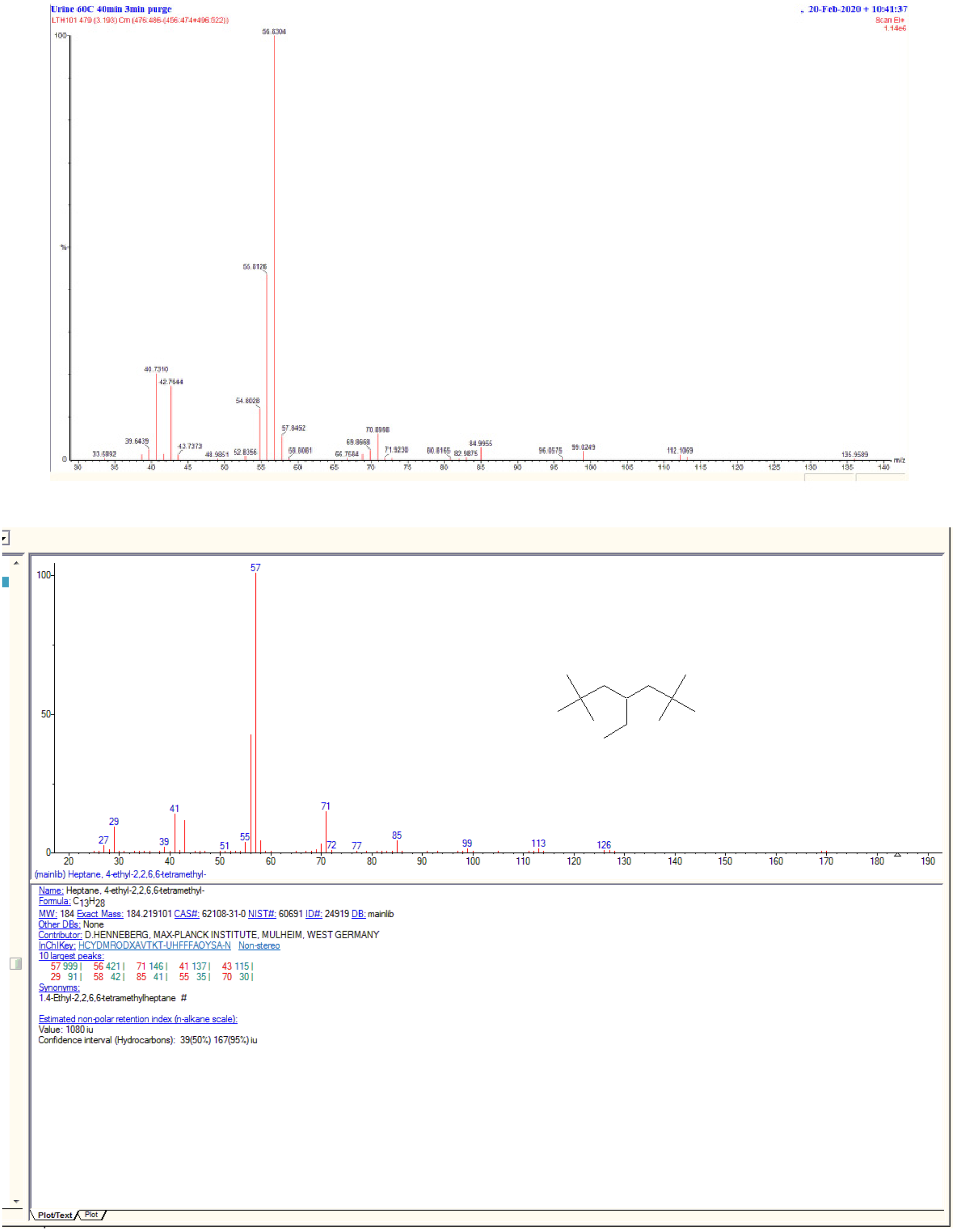

Figure S4. Experimental mass spectrum and best library match for the unknown compound at retention time 3.19 min.

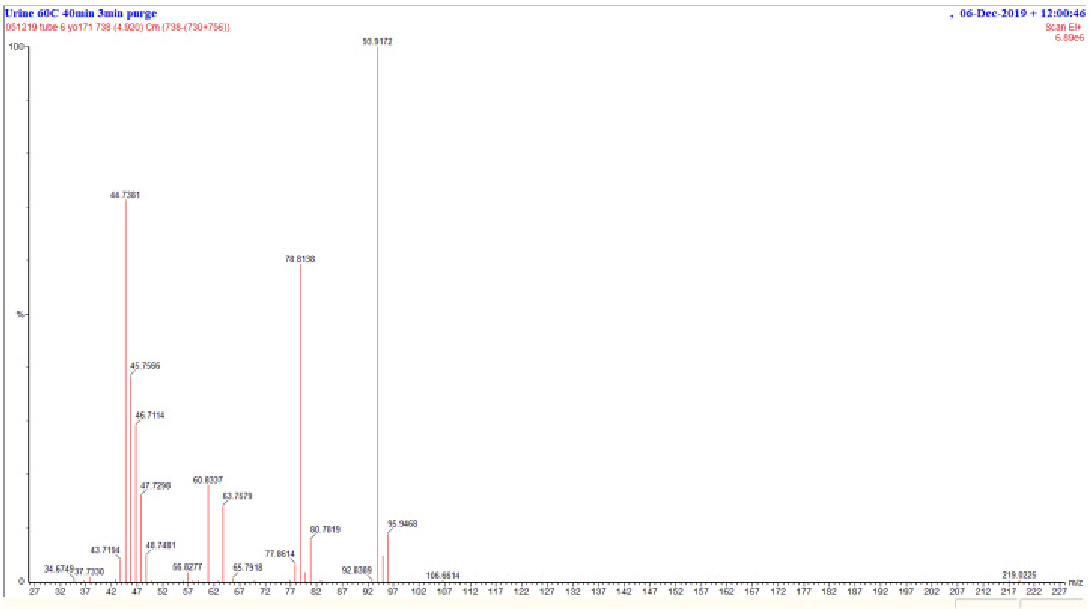

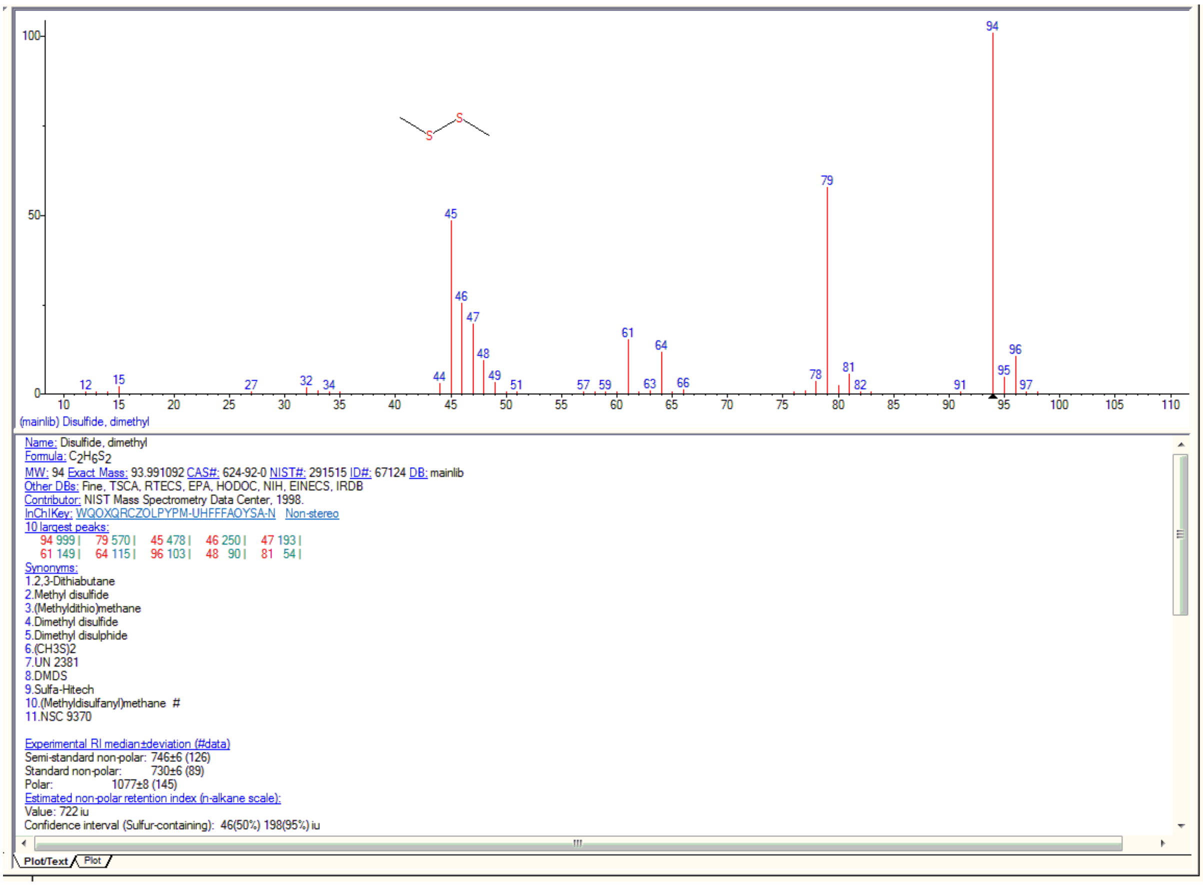

Figure S5. Experimental mass spectrum and library spectrum for the compound annotated as Dimethyldisulphide.

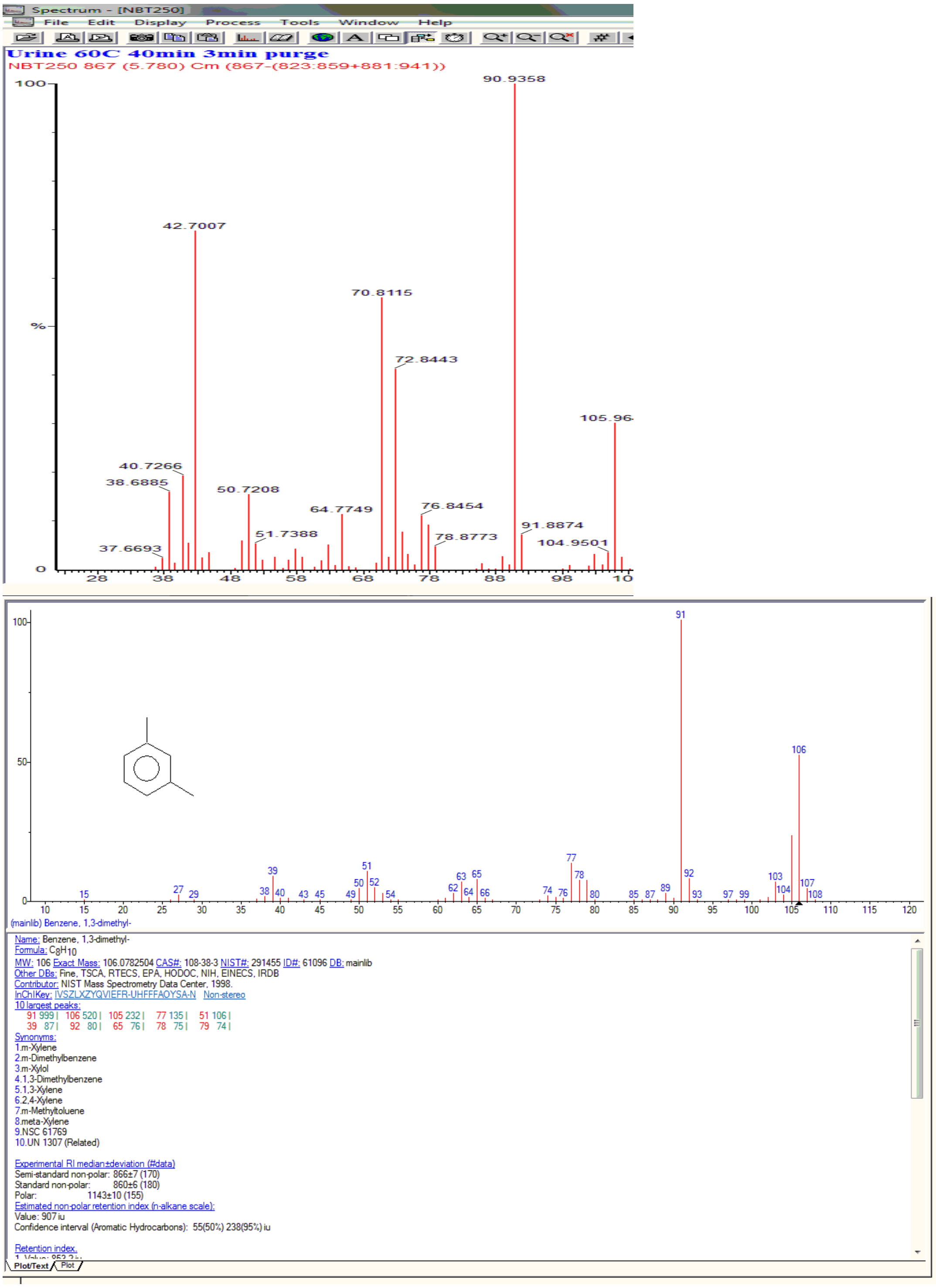

Figure S6. Experimental mass spectrum and library spectrum for the compound annotated as m-xylene.

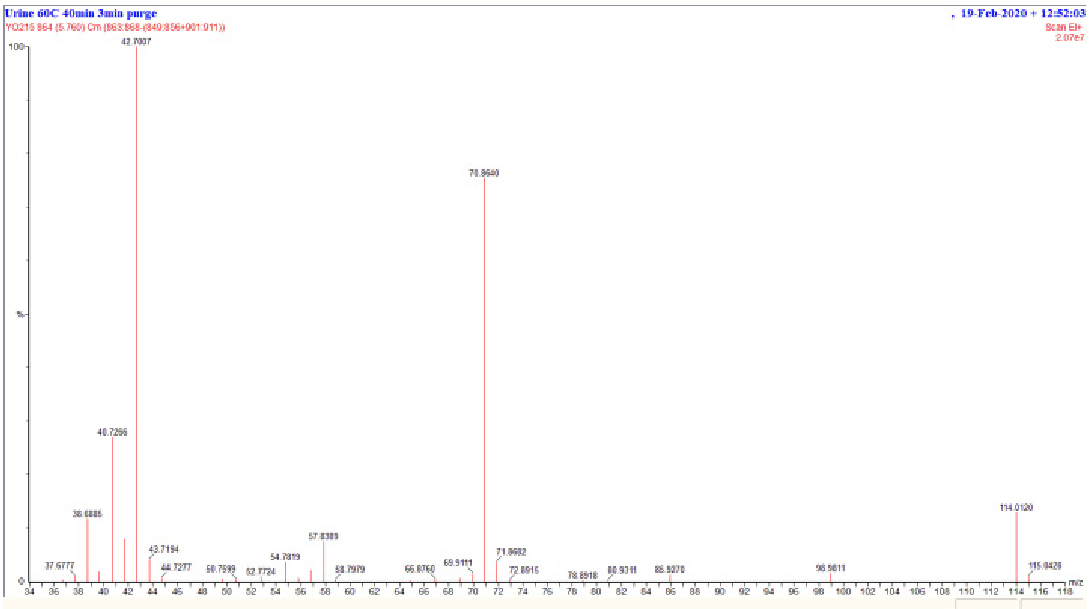

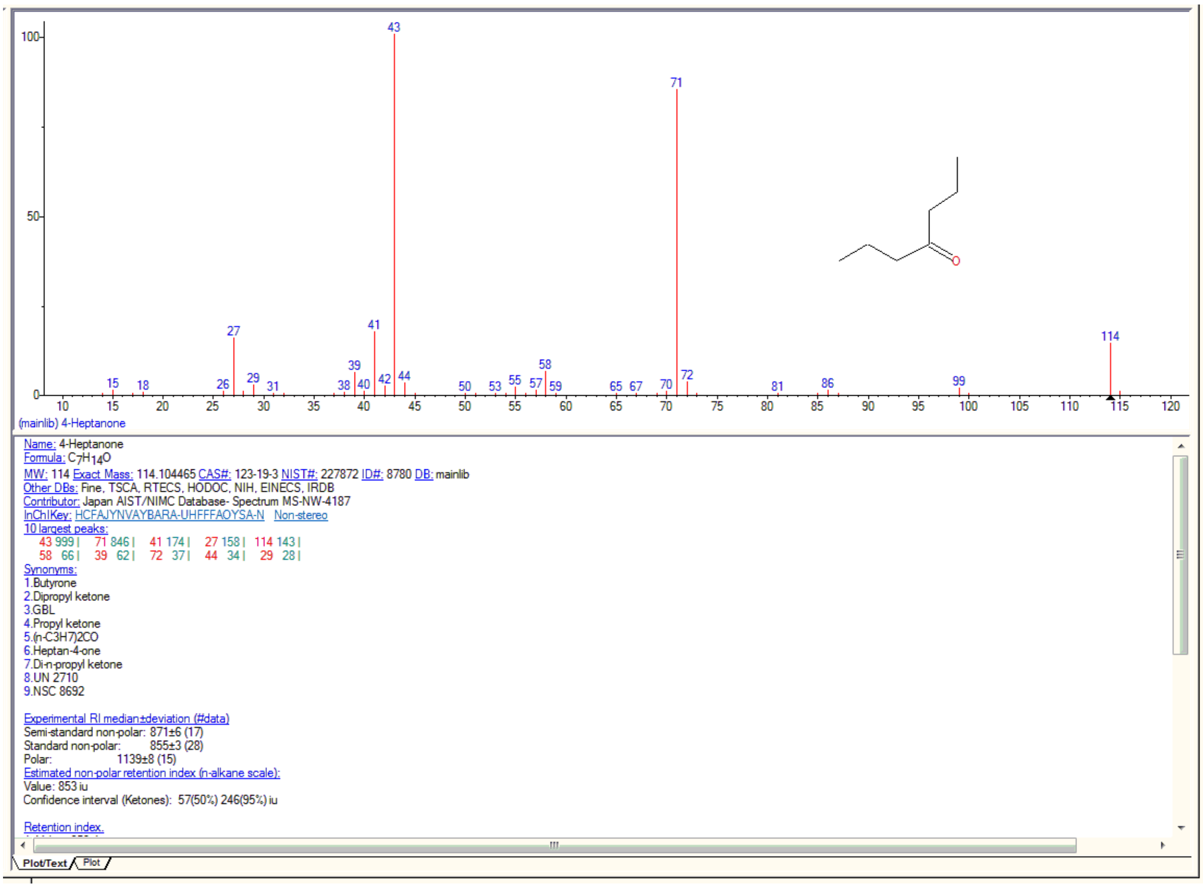

Figure S7. Experimental mass spectrum and library spectrum for the compound annotated as 4-heptanone.

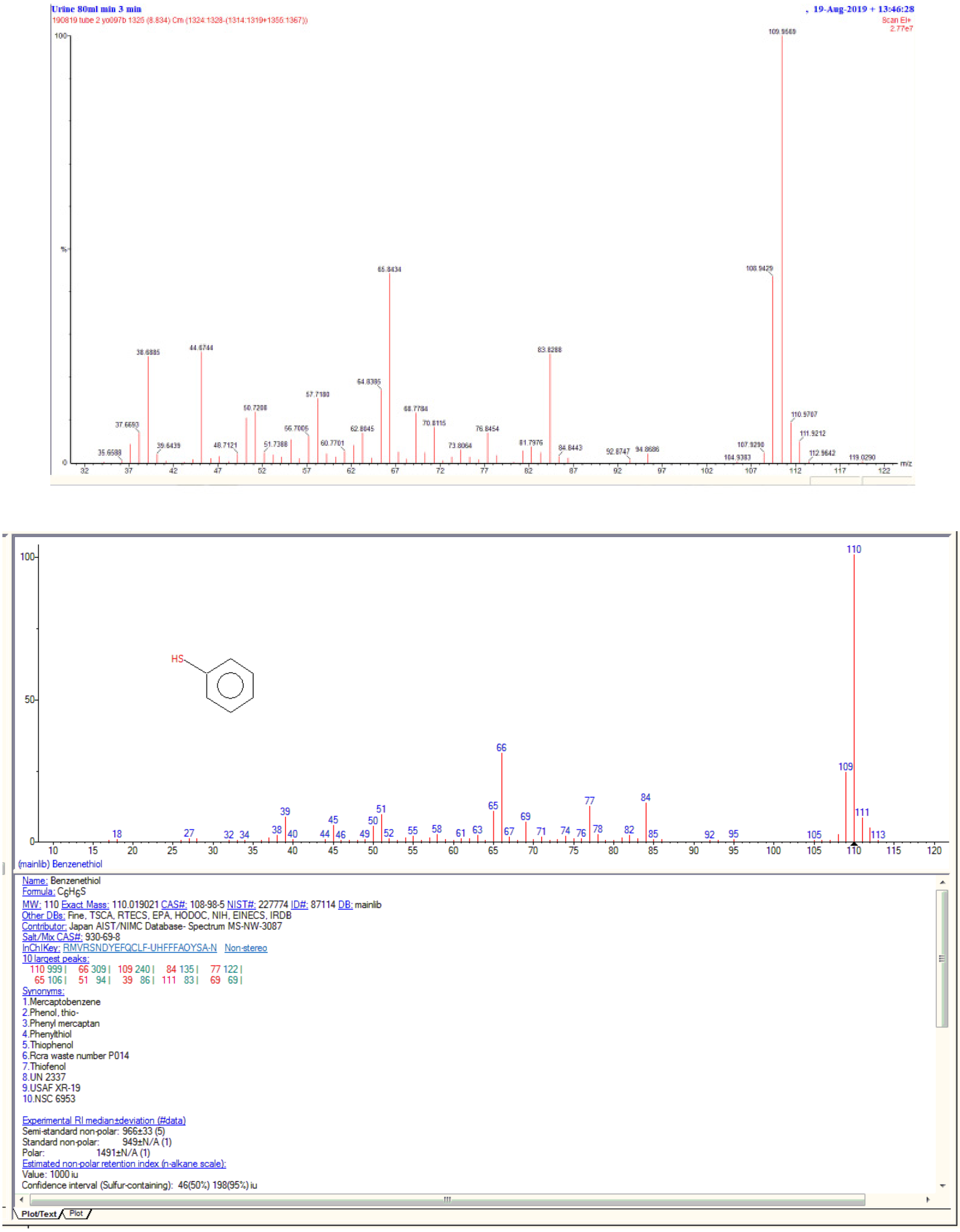

Figure S8. Experimental mass spectrum and library spectrum for the compound annotated as Benzenethiol.

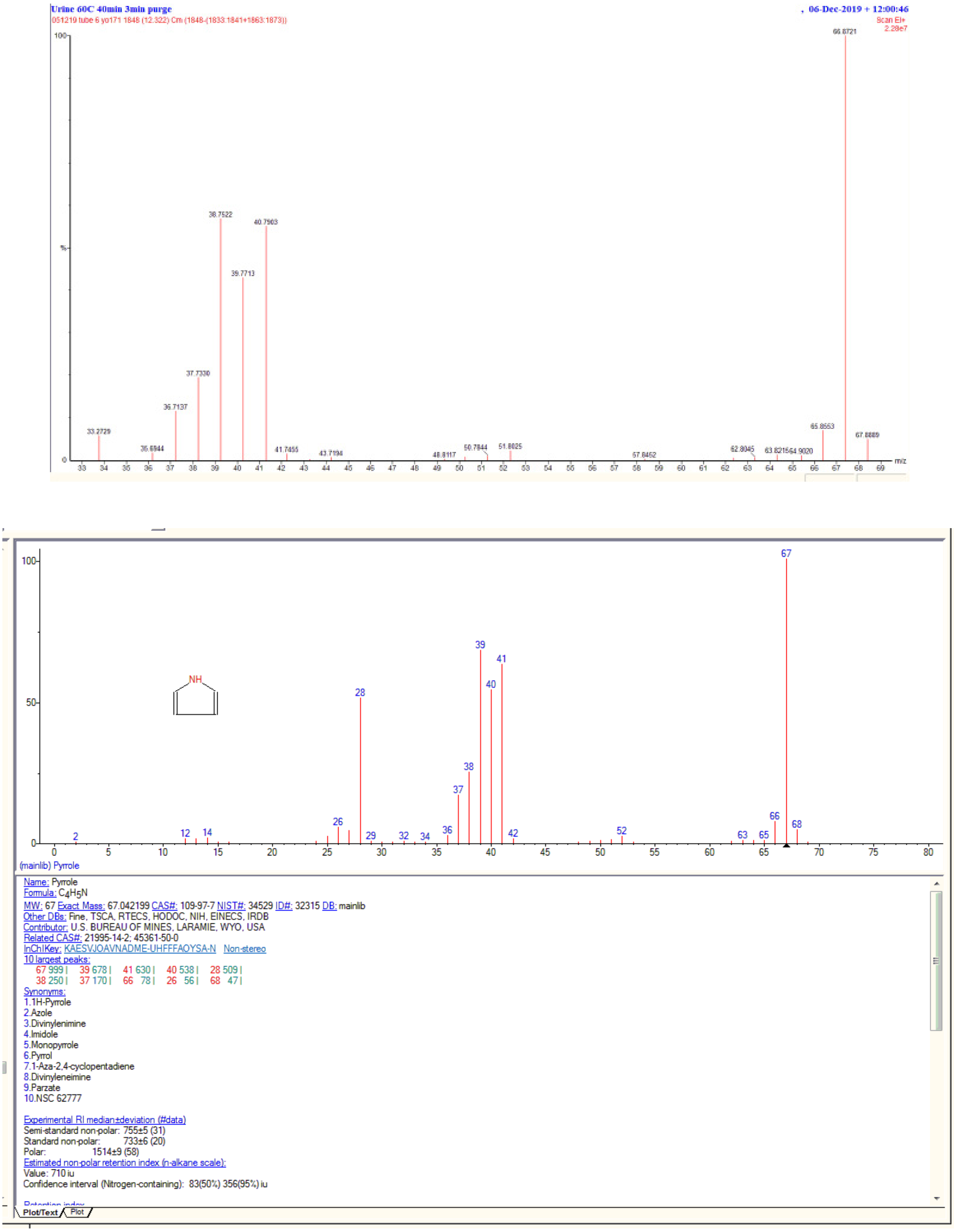

Figure S9. Experimental mass spectrum and library spectra for compound annotated as Pyrrole.

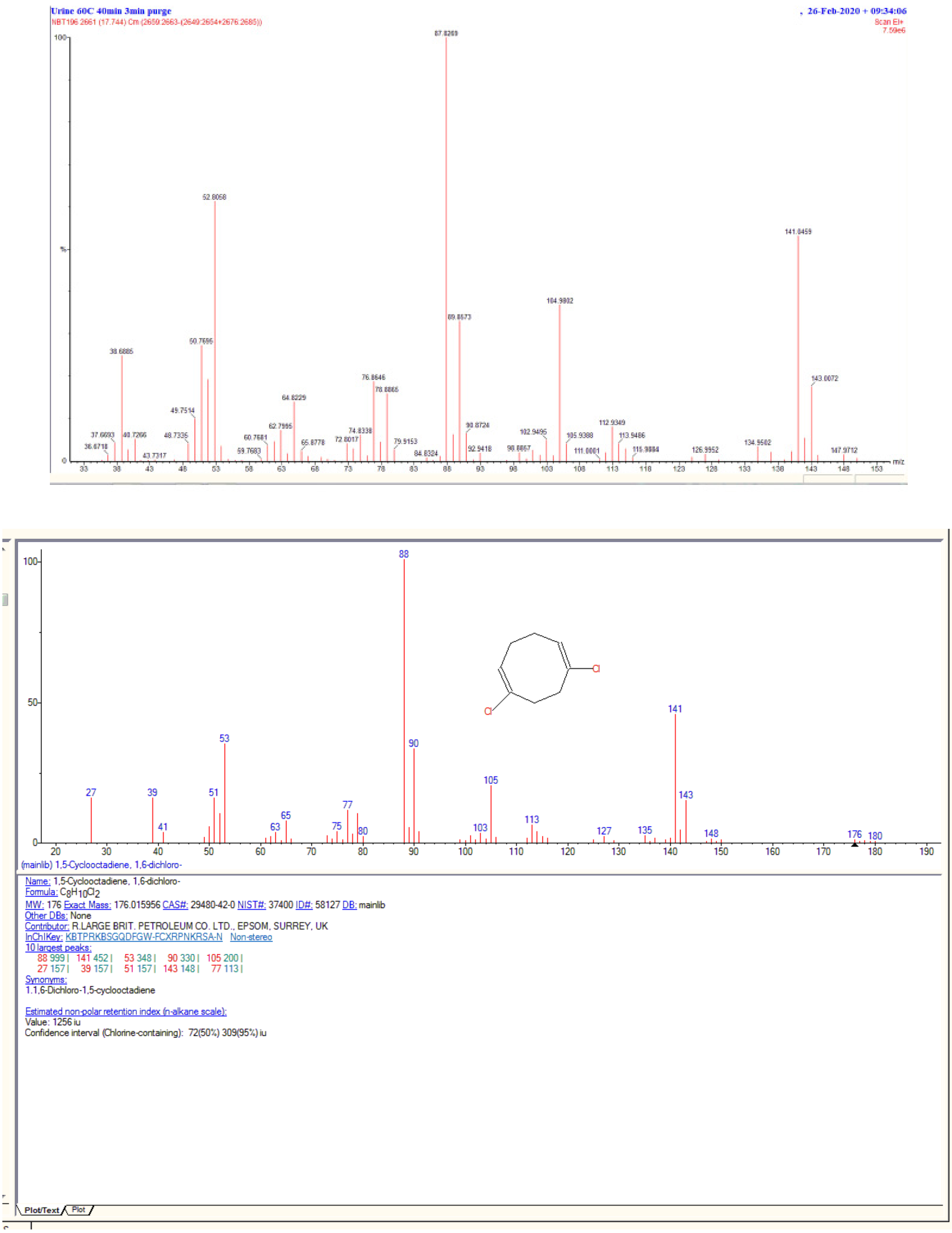

Figure S10. Experimental mass spectrum and library spectra for the compound annotated as 1,6-dichloro-1,5-cyclooctadiene.

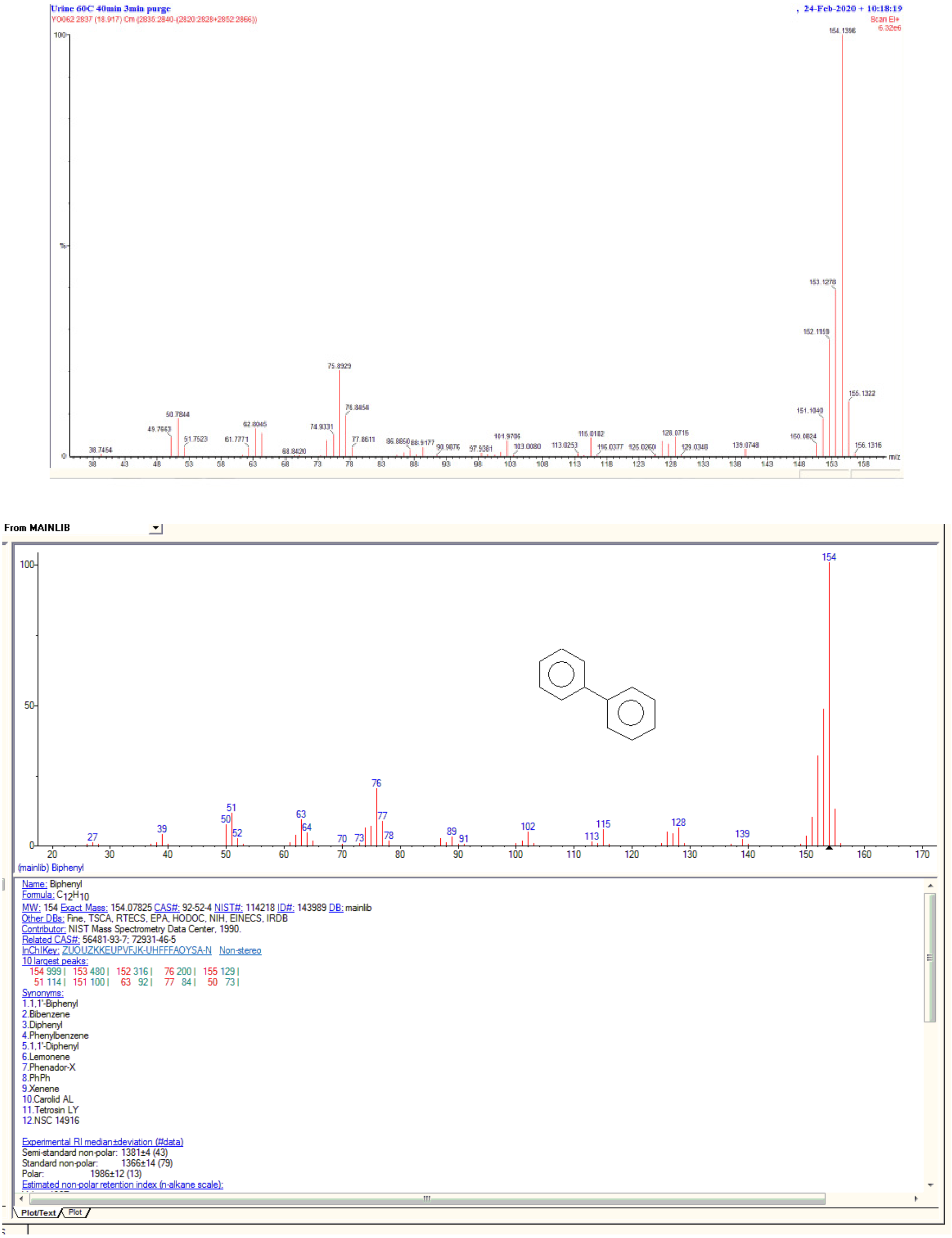

Figure S11. Experimental mass spectrum and library spectrum for compound annotated as Biphenyl.

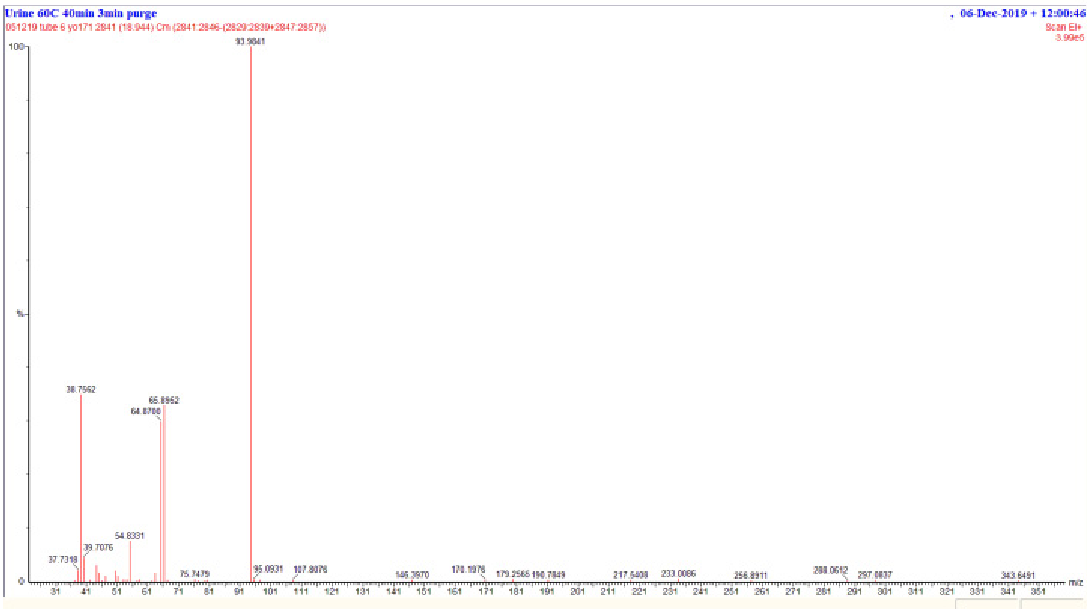

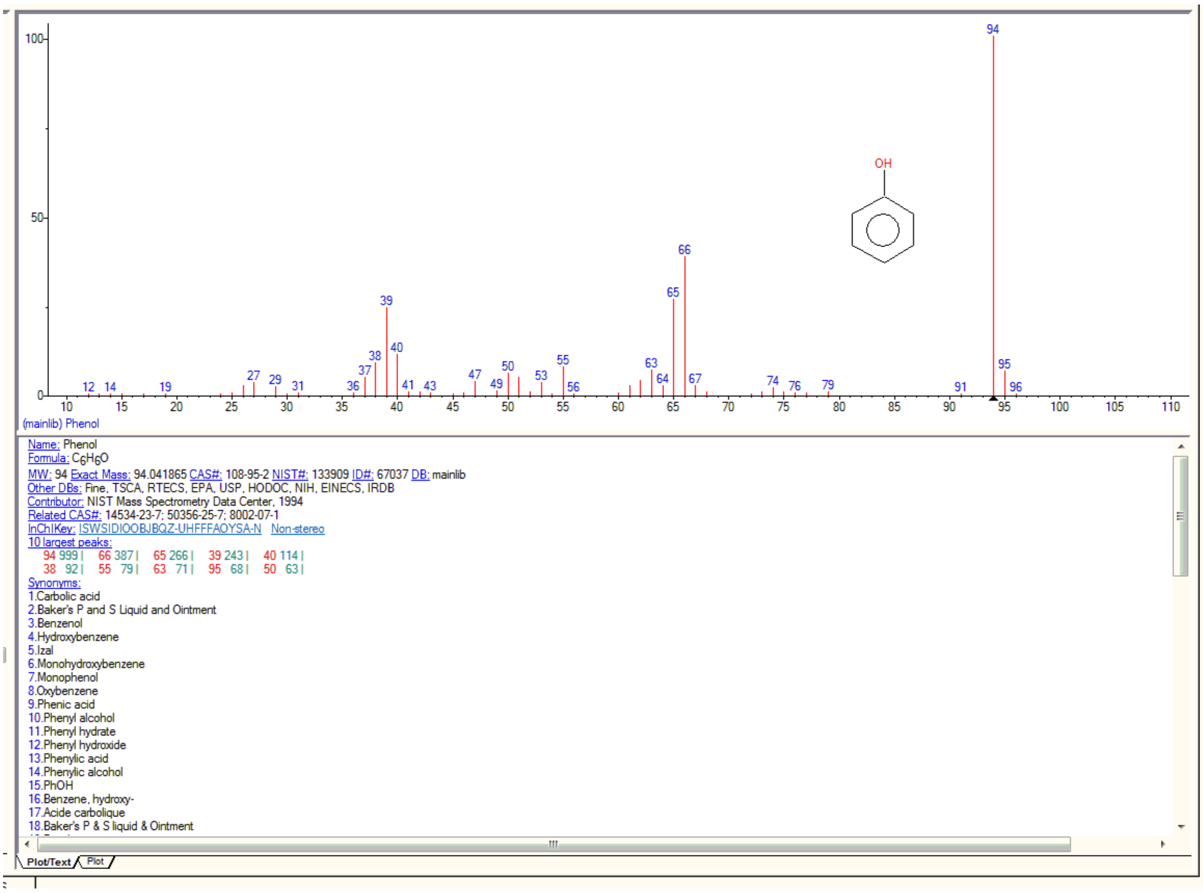

Figure S12. Experimental mass spectrum and library spectra for the compound annotated as Phenol.

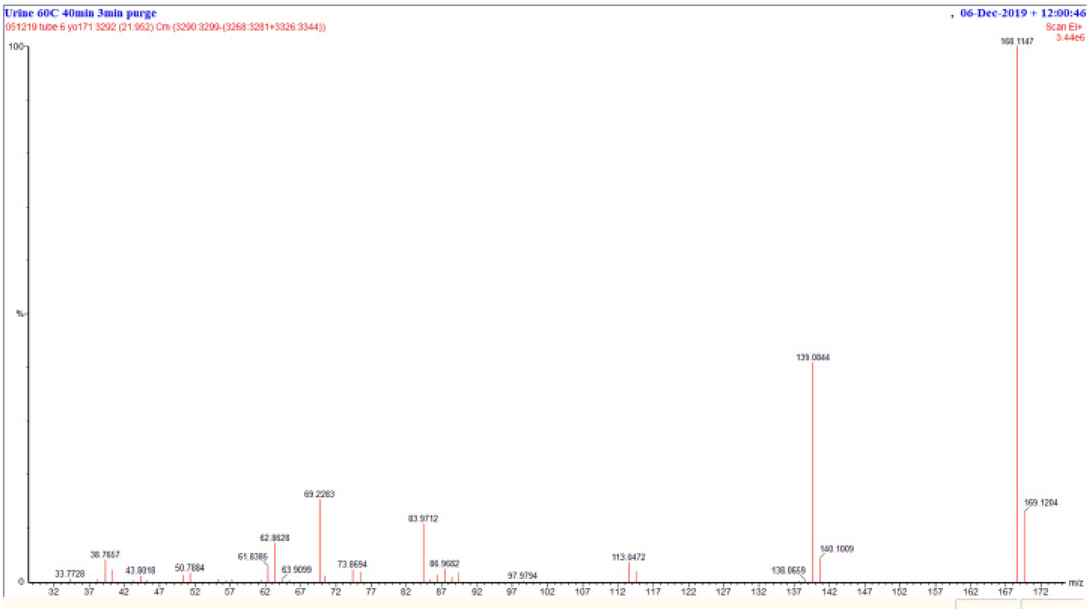

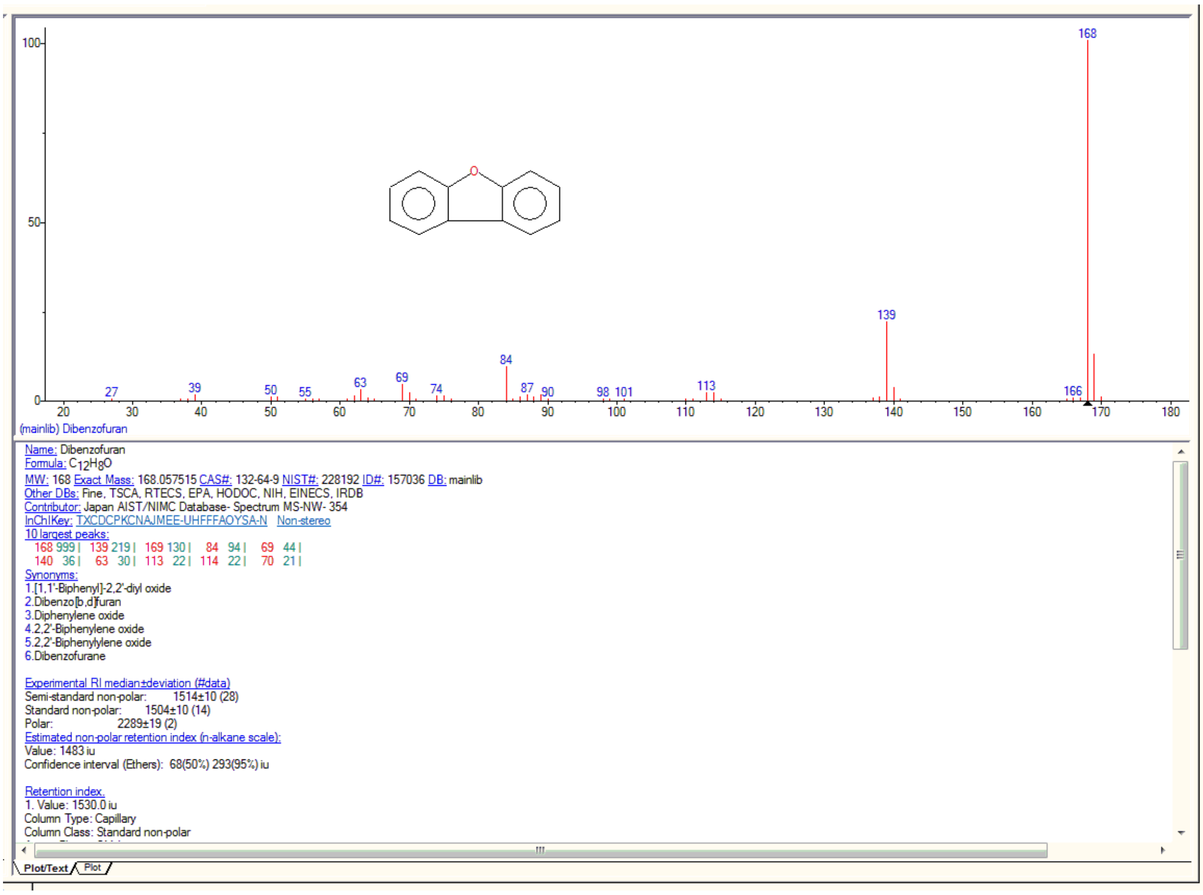

Figure S13. Experimental mass spectrum and library spectra for the compound annotated as Dibenzofuran.

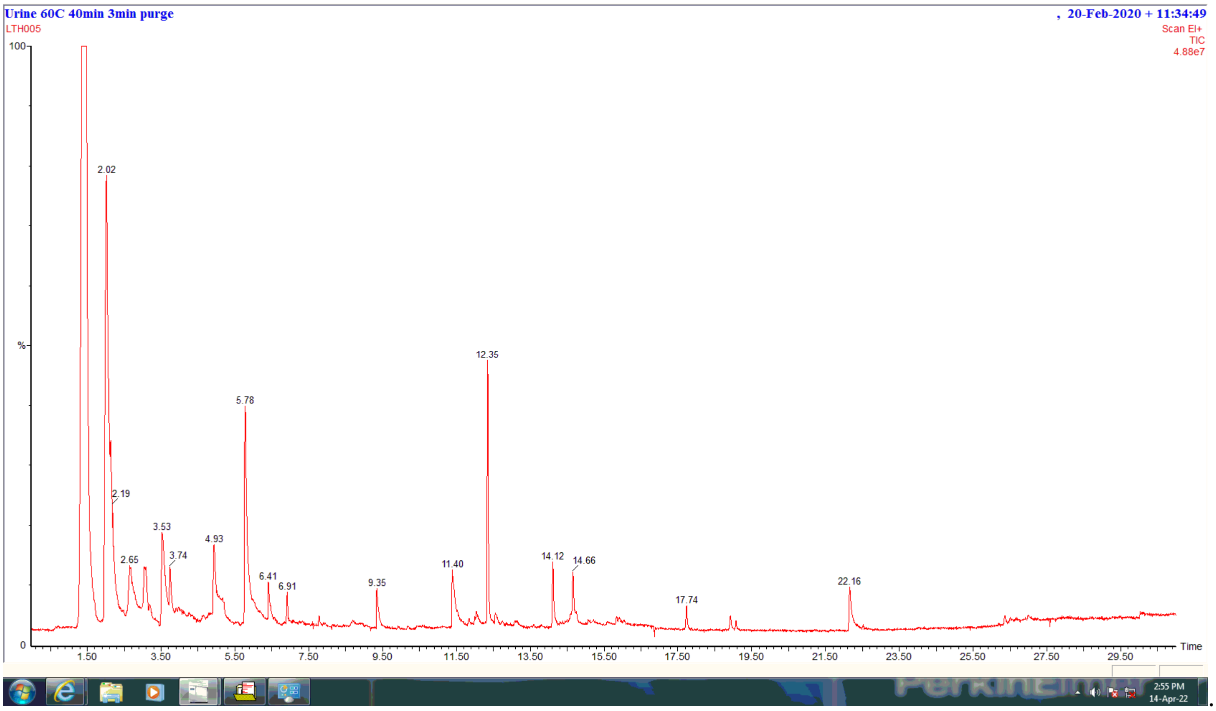

Figure S14. Chromatogram cancer.

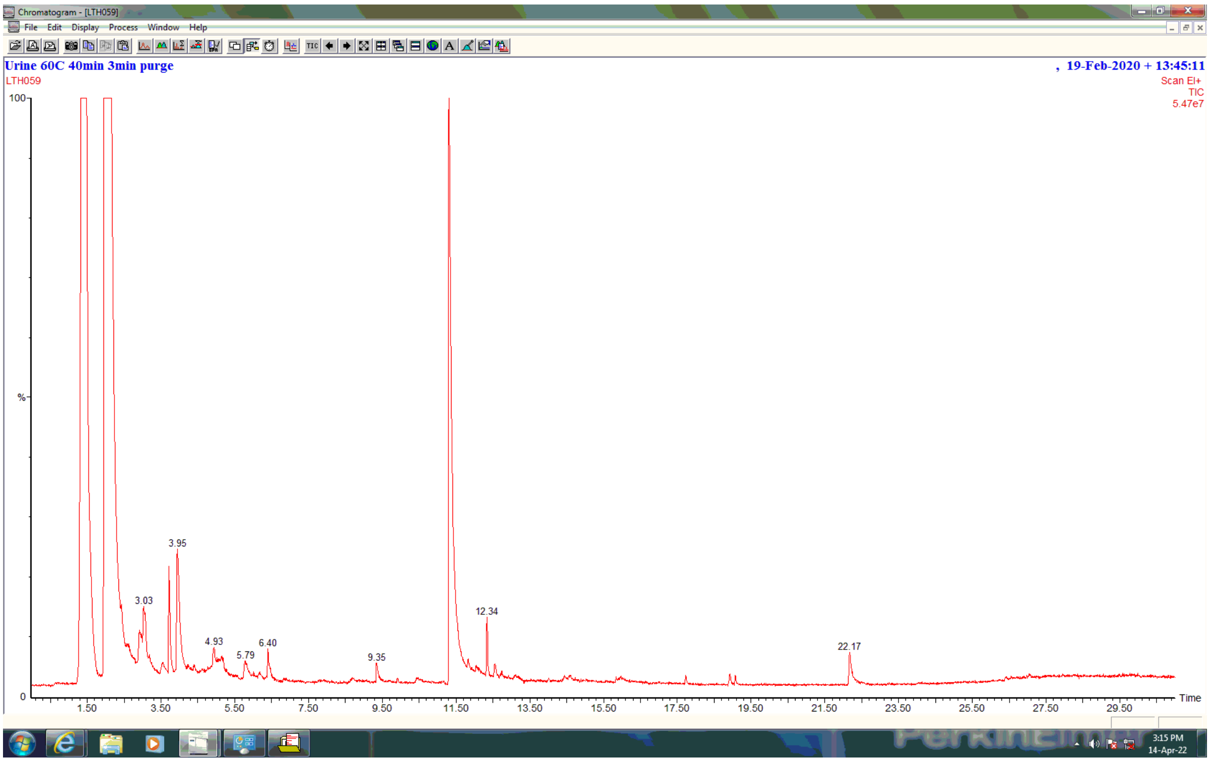

Figure S15. Chromatogram polyps.

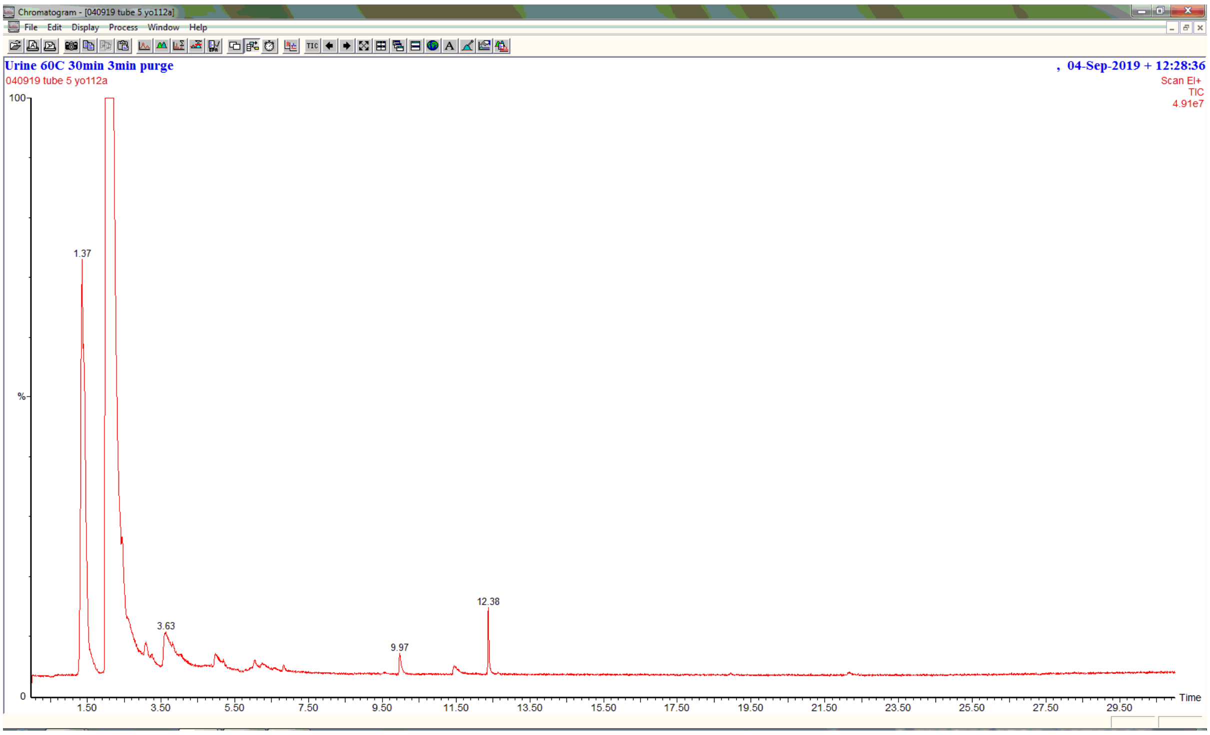

Figure S16. Chromatogram control.

References

1. Porto-Figueira, P.; Pereira, J.A.; Câmara, J.S. Exploring the potential of needle trap microextraction combined with chromatographic and statistical data to discriminate different types of cancer based on urinary volatomic biosignature *Anal. Chim. Acta* **2018**, *1023*, 53–63. https://doi.org/10.1016/j.aca.2018.04.027.
2. Porto-Figueirab, P.; Pereira, J.; Miekisch, W.; Câmara, J.S. Exploring the potential of NTME/GC-MS, in the establishment of urinary volatomic profiles. Lung cancer patients as case study. *Sci. Rep.* **2018**, *8*, 13113.
3. Arasaradnam, R.P.; McFarlane, M.J.; Ryan-Fisher, C.; Westenbrink, E.; Hodges, P.; Thomas, M.G.; Chambers, S.; O'Connell, N.; Bailey, C.; Harmston, C.; et al. Detection of Colorectal Cancer (CRC) by Urinary Volatile Organic Compound Analysis. *PLoS ONE* **2014**, *9*, e108750. https://doi.org/10.1371/journal.pone.0108750.
4. Wen, Q.; Boshier, P.; Myridakis, A.; Belluomo, I.; Hanna, G.B. Urinary volatile organic compound analysis for the diagnosis of cancer: A systematic literature review and quality assessment. Metabolites 2020, 11, 17–31
5. Huang, J.; Kumar, S.; Abbassi-Ghadi, N.; Španěl, P.; Smith, D.; Hanna, G.B. Selected Ion Flow Tube Mass Spectrometry Analysis of Volatile Metabolites in Urine Headspace for the Profiling of Gastro-Esophageal Cancer. *Anal. Chem.* **2013**, *85*, 3409–3416.
6. Opitz P.; Herbarth, O. The volatilome–investigation of volatile organic metabolites (VOM) as potential tumor markers in patients with head and neck squamous cell carcinoma (HNSCC) *J. Otolaryngol.—Head Neck Surg.* **2018**, *47*, 42. https://doi.org/10.1186/s40463-018-0288-5.
7. Ruiz Brandao da Costa, B.; Spinosa De Martinis, B. Analysis of urinary VOCs using mass spectrometric methods to diagnose cancer: A review. *Clin. Mass Spectrom.* **2020**, *18*, 27–37.
8. Silva, C.L.; Perestrelo, R.; Silva, P.; Tomás, H.; Câmara, J.S. Implementing a central composite design for the optimization of solid phase microextraction to establish the urinary volatomic expression: A first approach for breast cancer. *Metabolomics* **2019**, *15*, 64. https://doi.org/10.1007/s11306-019-1525-2.
9. Riordan, H.D.; Nancy Bramhall, B.S.; Neathery, S.; AMT M. Urine pyrroles in patients with cancer. *J. Orthomol. Med.* **2003**, *18*, 41–42.
